# A parallel program for the simulation of flooding

**DOI:** 10.1038/s41598-025-06868-z

**Published:** 2025-07-02

**Authors:** Anurak Busaman, Rhysa McNeil, Somporn Chuai-Aree, Mayuening Eso

**Affiliations:** 1 Department of Mathematics and Computer Science, Faculty of Science and Technology, Prince of Songkla University, Muang, Pattani Thailand; 2 Centre of Excellence in Mathematics, CHE, Si Ayutthaya Rd., Bangkok, 10400 Thailand

**Keywords:** Flood simulation, Finite volume method, Shallow water equations, Parallel algorithm, Automatic domain updating method, Environmental sciences, Natural hazards, Mathematics and computing

## Abstract

Accurate flood simulations are essential for effective prevention but they can be computationally slow and expensive, especially in large-scale scenarios. This can limit their use in time-critical situations. To solve this problem, this study aims to enhance the speed of numerical methods while maintaining accuracy in flood simulation results. A parallel algorithm is developed by applying the automatic domain updating method to solve the shallow water equations using a well-balanced, positivity-preserving first-order finite volume scheme. The parallel algorithm was designed with an index array to store the coordinates of cells in the computational domain that exclude unnecessary cells. The index array is divided and assigned to different cores, enabling parallel processing of each sub-domain. The developed parallel program was tested by simulating the water flow, compared with the results obtained in the literature, and applied to the Xe-Pian Xe-Namnoy dam break simulation in Laos. The computational times obtained by the proposed parallel program were compared with those of the serial program, which used only the automatic domain updating method without the parallel technique. The results show that the parallel program outperforms the serial program by reducing the computational time.

## Introduction

Accurate flood modeling and efficient computation is essential for risk assessment, disaster preparedness, and effective water management. Floods, whether caused by dam failures, river overflows, or storm surges, can lead to severe economic losses and loss of life^1^. Numerical models that simulate overland water flow provide crucial data for analyzing flood behavior, predicting inundation areas, and supporting real-time flood warning systems^2^. These models also play a key role in decision-making for evacuation planning and infrastructure design to mitigate flood impacts. Given the computational challenges of large-scale flood simulations, efficient numerical methods are necessary to ensure both accuracy and computational feasibility. This study focuses on developing a high-performance parallel algorithm to improve the efficiency of flood simulations while maintaining numerical accuracy, particularly in complex terrains and rapidly changing flow conditions.

The shallow water equations (SWEs) are fundamental for two-dimensional (2D) flood modeling, capturing complex flow dynamics through partial differential equations (PDEs) that require advanced numerical methods for efficient and accurate solutions. Among numerical methods, the finite volume method (FVM) is widely used for simulating water flow over complex topography due to its mass conservation properties and geometric flexibility. For instance, Elong et al.^3^ developed a 2D SWEs model using a FVM with a Godunov-type scheme to simulate flooding in the Tongo-Bassa Watershed, Cameroon. Their work demonstrated the capability of FVM in resolving real-world flood events over complex terrain. In another study, Yousif and Mohammed^4^ validated the suitability of 2D SWEs with FVM for urban flood modeling in Duhok city, accurately reproducing transient flow features and inundation patterns. In addition to accuracy, maintaining well-balanced properties is essential for stable simulations over uneven terrain. Peng et al.^5^ applied a well-balanced finite volume scheme to a 2D SWEs model, and showed that it significantly improved both stability and accuracy under complex flow conditions. The SWEs have been applied to model urban^6^, coastal^1^, river^1^, and dam-break^7^ floods, demonstrating their versatility. These examples demonstrate that 2D SWEs models are essential for capturing spatial variations in water depth and velocity, making them effective for simulating flood propagation in diverse terrains. Recognizing this, the present work focuses on developing and enhancing a 2D SWEs model based on the finite volume framework.

While FVM-based 2D SWEs models are highly effective at capturing complex flow dynamics, their use in real-world applications often faces challenges due to high computational demands. This is especially true for large or complex areas, such as urban regions with detailed topography, where high-resolution simulations are necessary. Since flood prevention requires to be done in time and quick decision-making, designing algorithms and numerical schemes for reducing computational time without losing much accuracy in the results is important.

One solution is to use parallel computing where the methods continuously divide the grid to each core processor follow features in the computing. Studies have explored parallel techniques, such as the parallelization of the finite volume method for simulating free-surface shallow water flows^8^. This approach distributes computations across multiple processors, significantly reducing the time needed to handle large and complex problems. A parallel implementation of shallow water solvers was developed for modeling overland flows^9^. High-performance computing (HPC) was specifically utilized to speed up simulations and manage larger datasets effectively. A fast simulation of large-scale floods has also been achieved using GPU-based parallel computing^10^. The method reduced computation time to levels suitable for real-time applications while efficiently processing detailed, high-resolution data. Furthermore, A simple algorithm was developed for the parallel computation of a grid-based one-dimensional distributed rainfall-runoff model^11^. This algorithm worked on both CPU and GPU systems, allowing for faster processing of high-resolution data and providing accurate results.

An alternative technique is to adjust (expand or shrink) the calculation area to exclude dry grid cells or unnecessary cells for computation. Studies used this technique such as the dynamic domain-defining method (dynamic DDM) in the development of GIS-based flood simulation software for flood-risk assessment^12^. The dynamic DDM divided the calculation area into several blocks. In the initial simulation, the calculation area was defined for the blocks nearby the river reaches. During the simulation, the calculation area was expanded to neighbouring blocks if a boundary cell of a block was flooded. However, this method still calculated some unnecessary cells for dry cells within blocks. Especially when there were a lot of dry cells inside the block. Moreover, this method can consume computational time for checking the boundary of each block in cases where there were a large number of block boundary cells.

An automatic domain updating (ADU) method for fast 2D flood-inundation modelling was developed by Tanaka et al.^13^. This method expanded the calculation area by including wet cells and their neighbours and checking only cells surrounding the flooded area. The ADU method and the dynamic DDM were used to simulate a bank-breaking scenario causing local flooding and a severe river overflow scenario. The results show that the ADU method outperforms the dynamic DDM. Additionally, Tanaka et al. explored the integration of ADU with parallel computing, achieving further performance gains for scenarios involving extensive domains. However, these efforts primarily utilized simplified formulations of the shallow water equations (SWEs), such as the local inertial approximation, and employed the finite difference method to solve the equations efficiently. These approaches offer computational advantages but may face challenges in capturing complex flow dynamics, especially in cases with rapid water level changes or irregular terrain, such as dam breaks.

Building upon this foundation, we propose integrating the ADU method with a parallelized solver for the full shallow water equations (Full SWEs). This approach addresses the limitations of simplified SWEs formulations by providing a more accurate representation of flow dynamics, including advection, pressure gradients, and bottom friction, which are critical in challenging scenarios like dam break simulations over irregular terrains. To achieve this with numerical stability and reliability, it is essential to incorporate a well-balanced, positivity-preserving finite volume scheme^14^. This scheme enhances numerical stability and accurately captures critical flow features, such as small water surface variations and steady states, while preventing nonphysical oscillations which is essential for maintaining accuracy in long-term simulations. This is particularly important in real-world flood scenarios where maintaining the correct balance between pressure forces and water movement is crucial. The proposed scheme combines advanced techniques, such as hydrostatic reconstruction, HLL flux, and semi-implicit friction calculations. Hydrostatic reconstruction enables accurate modelling of water flow over obstacles like levees, roads, and urban structures, preventing numerical errors that can arise in such conditions. Meanwhile, the employing of Harten-Lax-van Leer (HLL) flux^15^ and semi-implicit friction calculations^16^, the proposed method ensures higher numerical accuracy and stability, particularly in handling steep gradients and dry–wet transitions. Furthermore, we need a parallel algorithm that ensures efficient computation using required numerical schemes, supports dynamic domain resizing in the ADU method, and reduces communication overhead between cores by enabling direct access to neighboring cell data. This will be achieved through efficient domain management using domain decomposition and an index-based system. These methods are expected to enhance flood simulations, leading to more effective and reliable results.

Therefore, this study aims to develop a parallel program by applying the ADU method for solving the shallow water equations with the well-balanced positivity-preserving first-order finite volume scheme. The developed program is tested in various scenarios to validate its performance and accuracy. These include the simulation of flow on a parabolic bowl, which serves as an analytical benchmark, as well as dam break simulations under two distinct conditions: dry downstream and wet downstream. The method is further applied to the Xe-Pian Xe-Namnoy dam break flood simulation in Laos to demonstrate its applicability to real-world, complex flood scenarios. The results of the parallel program are then compared with those obtained from a serial program that uses only the ADU method without the parallel technique, highlighting the improvements in computational efficiency and accuracy achieved by the proposed approach.

## Methods

This study employs a well-balanced, positivity-preserving finite volume scheme to solve the shallow water equations for flood simulation. The methodology includes the governing equations of water flow, a numerical discretization method, and an automatic domain updating technique to dynamically adjust the computational domain for efficiency. Both serial and parallel algorithms are developed, with the parallel approach enhancing performance by distributing tasks across multiple processors. Each component is detailed in the following sections.

### The shallow water equations

The system of two-dimensional SWEs was used to represent the water flow behaviours. The derivation of the SWEs can be found in numerous text books^17,18^. The two-dimensional SWEs model combined with the friction term can be written as follows

1 $$\frac{\partial h}{{\partial t}} + \frac{\partial (uh)}{{\partial x}} + \frac{\partial (vh)}{{\partial y}} = 0$$

2 $$\frac{\partial (uh)}{{\partial t}} + \frac{{\partial (u^{2} h + \tfrac{g}{2}h^{2} )}}{\partial x} + \frac{\partial (vuh)}{{\partial y}} = - gh\frac{\partial z}{{\partial x}} - s_{x}$$

3 $$\frac{\partial (vh)}{{\partial t}} + \frac{\partial (uvh)}{{\partial x}} + \frac{{\partial (v^{2} h + \tfrac{g}{2}h^{2} )}}{\partial y} = - gh\frac{\partial z}{{\partial y}} - s_{y}$$

For these equations, $h$ is the water depth, $uh$ and $vh$ are water discharges per unit width, where $u$ and $v$ are velocity components in *x* and *y* directions. $z$ is topography height. $s_{x}$ and $s_{y}$ are the friction forces effected from the bottom roughness on the flow. The formulas are given by $s_{x} = gn^{2} uh\sqrt {u^{2} + v^{2} } h^{ - 4/3}$ and $s_{y} = gn^{2} vh\sqrt {u^{2} + v^{2} } h^{ - 4/3}$, where $n$ is the Manning’s roughness coefficient. For other variables, $g$ is gravitational acceleration, and $t$ is time. The first equation represents the conservation of mass, ensuring that the total volume of water remains constant over time. It accounts for variations in water depth as well as the horizontal fluxes of water within the computational domain. This equation is fundamental to accurately modelling water flow, as it governs how water levels change in response to inflows and outflows. The second and third equations describe the conservation of momentum in the *x* and *y* directions, respectively. These equations incorporate several key physical processes that influence water movement. First, they account for the transport of momentum due to the flow itself, a process known as advection. Additionally, they include the effects of pressure gradient forces, which arise from variations in water surface elevation and drive water movement from higher to lower elevations. Gravity plays a crucial role in directing flow downhill, while bottom friction, caused by interactions between the water and the underlying surface, opposes motion and influences flow velocity. Together, these equations provide a comprehensive framework for simulating the dynamic behaviour of water flow in natural. The equations can be written in a vector form as

4 $$\partial_{t} w + \partial_{x} f(w) + \partial_{y} g(w) = - z(w) - s(w)$$

In the vector form, $w = \left[ {h\,\,\,uh\,\,\,vh} \right]^{T}$ is the vector of the dependent variable. $f(w) = \left[ {uh\,\,\,u^{2} h + \tfrac{g}{2}h^{2} \,\,\,uvh} \right]^{T}$ and $g(w) = \left[ {vh\,\,\,vuh\,\,\,v^{2} h + \tfrac{g}{2}h^{2} } \right]^{T}$ are the flux functions in $x$ and $y$ directions, respectively. For the right-hand side of this vector equation, $z(w) = \left[ {0\,\,\,gh\partial_{x} z\,\,\,gh\partial_{y} z} \right]^{T}$ represents the gravity forces, while $s(w) = \left[ {0\,\,\,s_{x} \,\,\,s_{y} } \right]^{T}$ is the friction forces.

### The numerical method

To approximate the SWEs for numerical simulation, different discretization methods can be used, including the FDM, FVM, and FEM. FDM approximates derivatives using difference equations on a structured grid, making it computationally efficient but less suitable for handling complex geometries and ensuring conservation properties. FEM provides flexibility for irregular domains and higher-order accuracy but is computationally expensive for large-scale hydrodynamic simulations. FVM, on the other hand, applies conservation laws to control volumes, ensuring mass and momentum conservation while effectively handling shocks, discontinuities, and dry–wet transitions, making it well-suited for flood modeling. In this study, FVM is chosen for its balance between accuracy, computational efficiency, and robustness in handling complex terrain. Additionally, the Harten-Lax-van Leer (HLL) flux formulation is used to handle non-linear convective terms, ensuring numerical stability and accurate representation of flow dynamics.

To simulate water flow, the well-balanced positivity-preserving first-order finite volume scheme^14^ was used to find the solutions for the two-dimensional SWEs system. This scheme was chosen for its ability to reduce simulation run time by simplifying numerical processes, particularly in reconstruction and time integration. It simplifies reconstruction by using piecewise constant approximations, removing the need for complex slope calculations. Additionally, it replaces multi-stage TVD Runge–Kutta methods with explicit time-stepping schemes, minimizing the number of flux evaluations per step. These reductions streamline computations while preserving stability, making the method ideal for large-scale simulations like dam-break and flood modeling^19^. The components of the numerical scheme are described as follows:

#### Finite volume formulation

The finite volume method was performed on a simulation domain made of rectangular grid cells, where each cell is considered as a main control volume. The cell boundary is formed by the four direct walls surrounding it. By integrating the vector Eq. (4) over a cell domain having boundary $\tau$, together with applying the Green’s theorem, and dividing the equation by the cell area, the resulting formulation is derived.

5 $$\partial_{t} W_{i} + \frac{1}{\Delta A}\oint\limits_{\tau } {F \cdot n\,d\tau } + Z_{i} = - S_{i}$$

where, $i$ is the index of the cell. It was calculated from the cell position $(x,y)$ with $i = \omega y + x$, when $\omega$ is the width of the 2D computational grid. $\Delta A$ is the cell area. $W{}_{i},\,Z_{i}$ and $S{}_{i}$ represent the cell average value of $w$, $z(w)$ and $s(w)$, respectively, while $F = [f(w)\,\,\,g(w)]^{T}$ insteads the fluxes function vector. In this equation, $n$ is the unit outward normal vector of the cell boundary.

By doing based on the Audduse’s scheme^14^, the line integral combined with the gravity term is approximated by the summation of four numerical fluxes surrounding the cell.

6 $$\frac{1}{\Delta A}\oint\limits_{\tau } {F \cdot n\,d\tau } + Z_{i} \approx \frac{1}{\Delta A}\sum\limits_{k = 1}^{4} {\hat{F}_{k}^{t} \cdot n_{k} \,\Delta \tau_{k} }$$

when $\hat{F}_{k}^{t} \cdot n_{k} \,$ is the numerical fluxes, approximated by a chosen scheme, and $\Delta \tau_{k}$ is the size of the interface between the cell and its neighbor. $k$ is index of the interfaces. By substituting Eq. (6) into Eq. (5) and using Euler’s method for time discretization, the discrete form of the equation becomes:

7 $$W_{i}^{t + \Delta t} = W_{i}^{t} - \frac{\Delta t}{{\Delta A}}\sum\limits_{k = 1}^{4} {\hat{F}_{k}^{t} \cdot n_{k} \Delta \tau_{k} } - \Delta tS_{i}^{t}$$

where $\Delta t$ is the time step size.

#### Numerical fluxes

For the numerical fluxes, the chosen scheme is based on Harten, Lax and van Leer (HLL) flux^15^. This method has been widely applied in recent studies, such as Klingenberg et al.^20^ and Hwang and Son^21^. The numerical fluxes can be calculated at each boundary wall between the cell and its neighbors. The formula of the numerical fluxes can be written as the followings.

8 $$\hat{F}_{k}^{t} \cdot n_{k} = \frac{{\lambda_{{}}^{ + } \hat{F}(U_{k - }^{t} ) \cdot n_{k} - \lambda_{{}}^{ - } \hat{F}(U_{k + }^{t} ) \cdot n_{k} }}{{\lambda_{{}}^{ + } - \lambda_{{}}^{ - } }} + \frac{{\lambda_{{}}^{ + } \lambda_{{}}^{ - } (U_{k + }^{t} - U_{k - }^{t} )}}{{\lambda_{{}}^{ + } - \lambda_{{}}^{ - } }} + \hat{Z}(U_{k - }^{t} ) \cdot n_{k}$$

In this formula, $\hat{F}(U) = \left[ {f(U)\,\,\,g(U)} \right]^{T}$ is the numerical flux function, where $U_{k \pm }^{t} = \left[ {\hat{h}_{k \pm }^{t} \,\,\,(u\hat{h})_{k \pm }^{t} \,\,\,(v\hat{h})_{k \pm }^{t} } \right]^{T}$ is a dependent variable vector, according to the hydrostatic reconstruction^14^ given by

9 $$\hat{h}_{k \pm }^{t} = \max \left\{ {0,h_{k \pm }^{t} + z_{k \pm }^{{}} - \max \{ z_{k - }^{{}} ,z_{k + }^{{}} \} } \right\}$$

In this scheme, $\hat{Z}(U) = \left[ {z_{x} \,\,\,z_{y} } \right]^{T}$ is term to satisfy the balance of momentum flux and momentum gravity forces in the first order scheme, where $z_{x} = \left[ {0\,\,\, - \tfrac{g}{2}(\hat{h}_{k - }^{t} )^{2} \,\,\,0} \right]^{T}$ and $z_{y} = \left[ {0\,\,\,0\,\,\, - \tfrac{g}{2}(\hat{h}_{k - }^{t} )^{2} } \right]^{T}$. $\lambda_{{}}^{ + }$ and $\lambda_{{}}^{ - }$ are the wave speeds computed based on Kurganov et al.^22^ as follows

10 $$\lambda_{{}}^{ \pm } = \pm \max \left\{ { \pm q_{k - }^{t} \cdot n + \sqrt {g\hat{h}_{k - }^{t} } ,\,\, \pm q_{k + }^{t} \cdot n + \sqrt {g\hat{h}_{k + }^{t} } ,\,\,0} \right\}$$

when $q = \left[ {u,\,\,v} \right]^{T}$ is the vector of the velocities. In these equations, *k-* and *k* + are the special index of the values the interface *k,* within the cell *i* and its neighbor cell, respectively.

#### Friction forces calculation

A semi-implicit method^16^ was adopted for the friction forces, where the water discharge values obtained from the Eq. (7) is updated as follows.

11 $$uh_{i}^{t + \Delta t} \leftarrow \frac{{uh_{i}^{t + \Delta t} }}{{1 + \Delta tgn^{2} \sqrt {(u_{i}^{t} )^{2} + (v_{i}^{t} )^{2} } (h_{i}^{t + \Delta t} )^{ - 4/3} }}$$

and

12 $$vh_{i}^{t + \Delta t} \leftarrow \frac{{vh_{i}^{t + \Delta t} }}{{1 + \Delta tgn^{2} \sqrt {(u_{i}^{t} )^{2} + (v_{i}^{t} )^{2} } (h_{i}^{t + \Delta t} )^{ - 4/3} }}$$

where the sign ‘$\leftarrow$’ mean that the left-hand side value is changed to the right-hand side value. These updating were performed in order to ensure stability of the solution.

#### Stability condition

To ensure stable solutions, the time step $\Delta t$ in the Eq. (7) was determined using the Courant Friedrich Lewy (CFL) condition, as described in Hargen^23^:

13 $$\Delta t = 0.5\frac{{\Delta \tau_{\min } }}{{\lambda_{\max } }}$$

when, $\lambda_{\max }$ represents the maximum absolute value of all wave speeds obtained during the water flow simulation at a given time. Additionally, $\Delta \tau_{\min } = \min \{ \Delta x,\Delta y\}$, where $\Delta x$ and $\Delta y$ denote the sizes of the cell interfaces in $x$ and $y$ directions, respectively.

#### Initial and boundaries condition

The initial conditions were defined based on the experimental setup, which are water depths and topography heights. Initially, the water discharges were set to zero. At each time step during the simulation, two types of boundary conditions, which are the closed and the open were chosen to implement in the simulation. As the simulation progressed, two types of boundary conditions, closed and open, were implemented at each time step. The closed boundary condition was defined as follows:

14 $$W_{b}^{t} = 0\,,\,\,z_{b} = z_{M}$$

where the subscript $b$ refers to ghost cells outside the grid domain, and $z_{M}$ represents a large constant value for the topography height. To impose the open boundary condition, the following formulation was used:

15 $$W_{b}^{t} = W_{Nb}^{t} \,,\,\,z_{b} = z_{Nb}$$

when the subscript $Nb$ refers to a neighbor cell of the boundary cell *b.*

### The automatic domain updating method

In water flow simulations, if a cell and its neighboring cells are all dry, the fluxes of flow are zero. Consequently, the cell-dependent value is also zero, and the exact solution for the cell is already known, making numerical computation unnecessary. To reduce computational time, the ADU method is applied in this study. The ADU method skips solving the water flow equations in unnecessary cells during the simulation.

At the beginning of the simulation, the ADU method defines the computational domain by extracting only the necessary cells for computation, including wet cells and their immediate neighbors. In this step, all cells are checked based on their wet-dry conditions. A cell *i* and its neighbor cells are included in the computational domain if the cell *i* is wet, i.e., $h_{i}^{t} > 0$. This approach ensures that cells with even minimal water are treated as wet, thereby maintaining flux stability and guaranteeing that the results obtained using the ADU method are exact to those from full-domain computations. Before adding a cell to the domain, it is verified that the cell is not already included, thus avoiding errors from redundant calculations. Subsequently, dry cells within the computational domain are re-extracted to define the boundaries of the wet area.

During the simulation, only the boundary cells of the wet area are checked to expand the computational domain. The boundary is updated to include newly dry cells and their surrounding wet cells. This approach of updating the computational domain by checking only the boundary cells significantly reduces computational time. The ADU method is applied after obtaining the solution of the Eq. (5) via Euler’s method to determine a new computational domain for each iteration.

### The serial algorithm

**Fig. 1:**
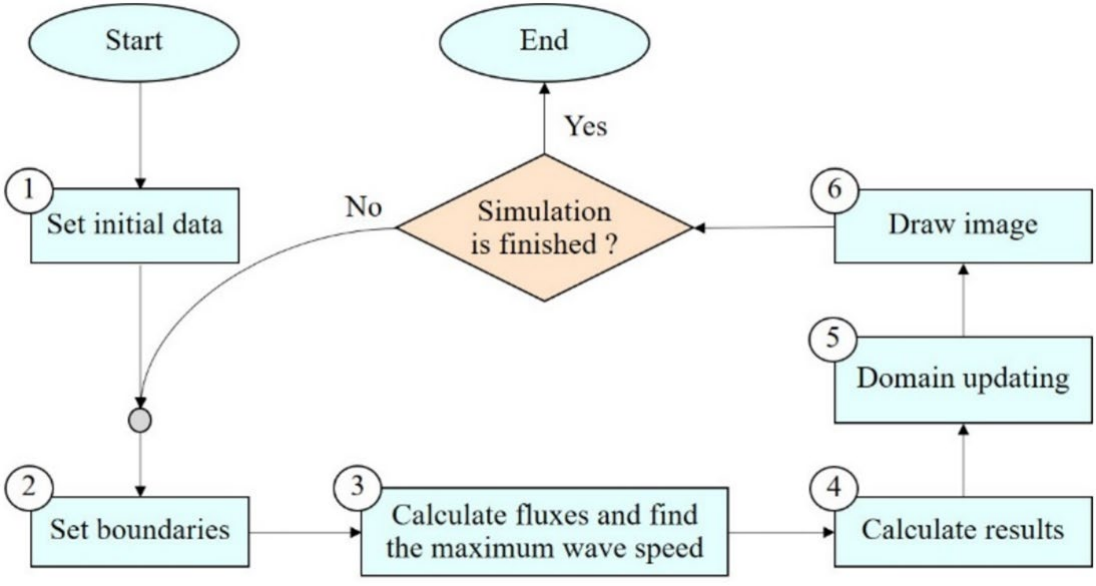
The flowchart of the serial program for solving the water flow equations by using the ADU method.


In this section, the algorithm of the serial program based on the ADU method (without parallel algorithm) for solving the water flow equations are presented. The algorithm can be represented in the form of a flowchart as illustrated in Fig. 1. The details for each step are described below:

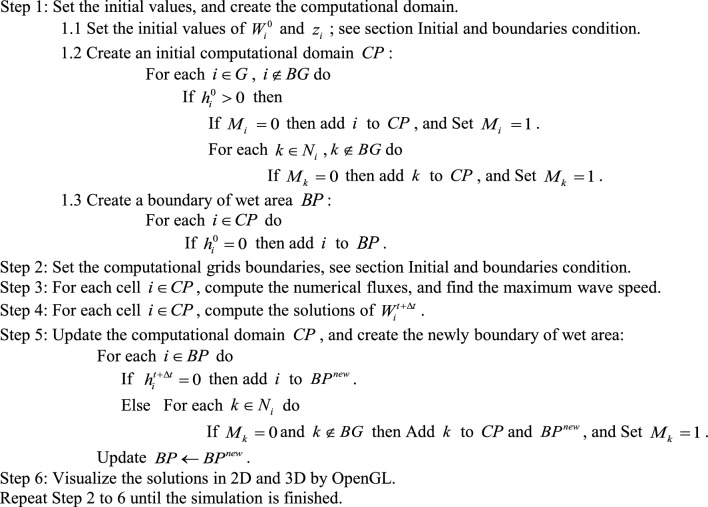


In here, $G$ and $BG$ are the entire simulation domain and its boundary, respectively, while $CP$ and $BP$ are the set of point indices in the computational domain and the boundary of wet area, respectively. $N_{i}$ is the set of neighbor cells of the cell $i$. $M_{k}$ is the checker that the cell $k$ is in the computational domain ($M_{k} = 1$) or not ($M_{k} = 0$).

### The parallel algorithm

**Fig. 2:**
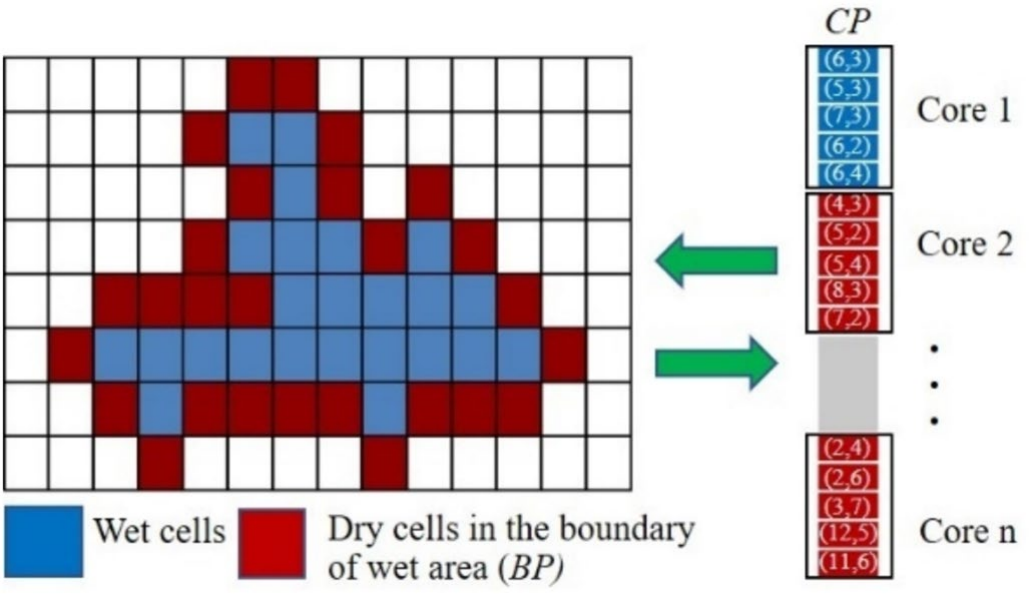
The data grid and the array of indices that store the coordinates of cells in the computational domain for the parallel algorithm.

This section presents the algorithm of the parallel program based on the ADU method for solving the water flow equations. To implement parallel computation combined with the ADU method, the computational domain is partitioned and distributed across processor cores, enabling simultaneous calculations. The parallel algorithm was developed using an array of indices to store the coordinates of cells in the computational domain, as illustrated in Fig. 2. During the simulation, this array is divided among the cores to compute solutions for different sub-areas in parallel. The numerical fluxes and solutions for each cell are calculated by retrieving the variable values of the specified cell and its neighbors from the data grid.

**Fig. 3:**
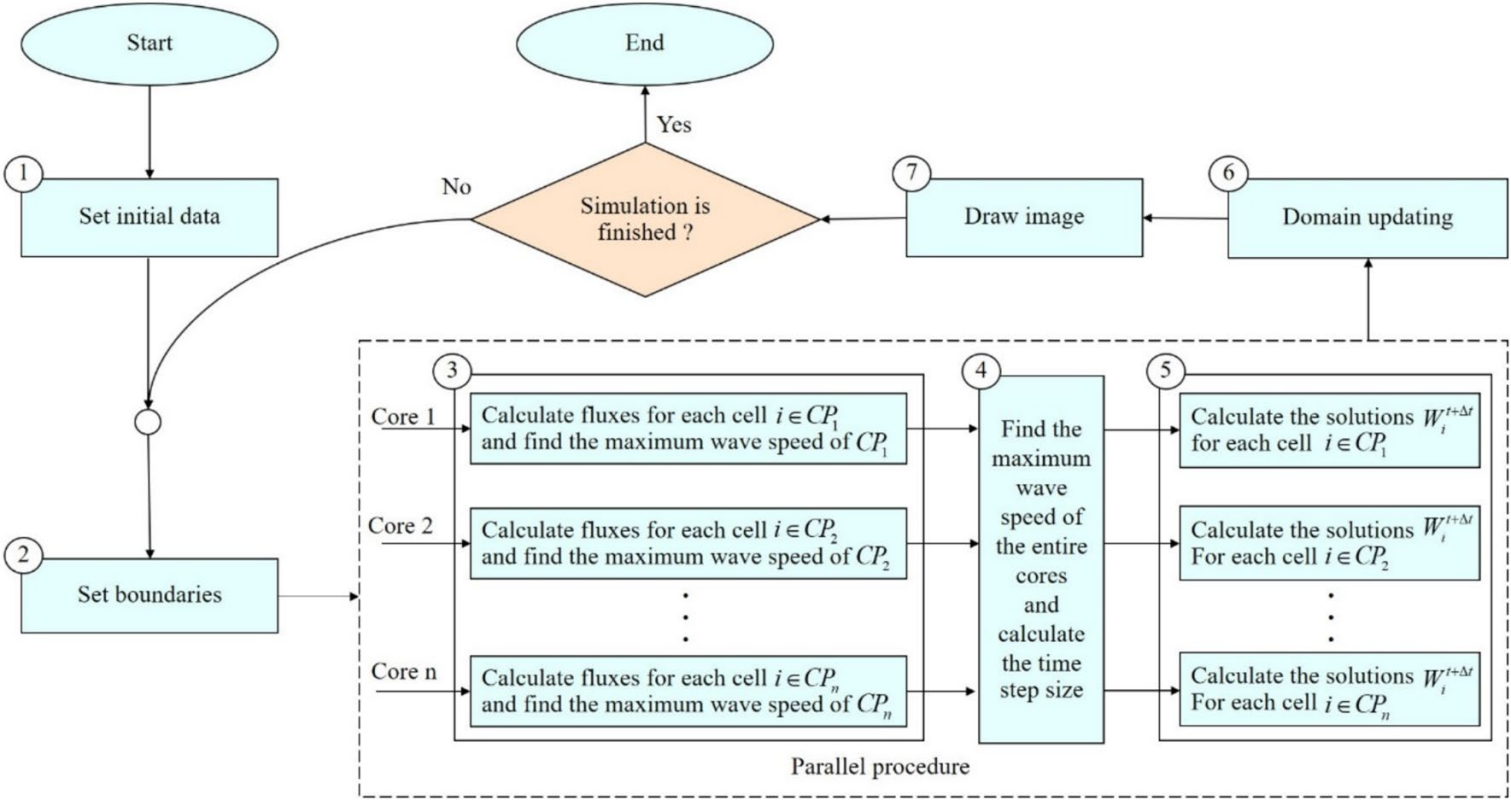
The flowchart of the parallel program for solving the water flow equations by using the ADU method.


The algorithm of the parallel program for solving the water flow equations can be represented in the form of a flowchart as illustrated in Fig. 3. This algorithm applies parallel computing to specific steps of the serial program, specifically the calculation of numerical fluxes and solutions (steps 3 and 4 in Fig. 1), and adds an additional step to determine the maximum wave speed obtained across all cores. The details of each step are described below:

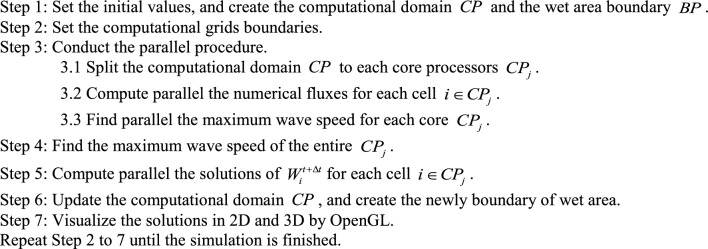


In here, $CP_{j}$ is the set of point indices in the computational domain of the core processor $j$.

### Numerical results

We illustrated the numerical simulations to demonstrate the accuracy and effectiveness of the proposed parallel algorithm for the simulation of shallow water flow. The experiments consisted of the simulation of a 2D parabolic bowl, the dam break simulation in the dry case, and the dam break simulation in the wet case. In the last testing, the parallel algorithm was applied for the simulation of a dam break on natural topography, the simulation of the assumed event of the Xe-Pian Xe-Namnoy dam break in Laos. The results were calculated by using computer notebook with CPU AMD Ryzen5-4500U, 2.38 GHz, RAM DDR4-8 GB.

#### 2D Parabolic bowl simulation

The numerical simulation of a water flow on a 2D parabolic bed slope is performed using the parallel algorithm and then compared with the results of study using analytic solutions^24^. The 2D parabolic bowl test case is a variant of Thacker’s solution^25^ with Manning’s friction term. For the domain $L \times L$ meters, the 2D parabolic bed topography is given by

16 $$z(x,y) = \frac{{h_{0} }}{{a^{2} }}\left[ {\left( {x - \frac{L}{2}} \right)^{2} + \left( {y - \frac{L}{2}} \right)^{2} } \right]$$

where $h_{0}$ and $a$ are positive constants. For the analytic solution of the test case, this was obtained based on the relationship between the bed friction parameter $\tau$ and a peak amplitude parameter $p = \sqrt {8gh_{0} }$. For $\tau < p$, the analytic solution for the water depths $h\left( {x,y,t} \right)$ and the velocities $u\left( t \right)$ and $v\left( t \right)$ can be written as follows.

17 $$\begin{aligned} h\left( {x,y,t} \right) = & h_{0} - \frac{1 \, }{{2g}} \, B^{2} e^{ - \tau t} - \frac{1 \, }{g} \, Be^{{ - \frac{\tau t}{2}}} \left( {\frac{\tau }{2}sin \, st \, + \, s\,cos \, st} \right)\left( {x - \, \frac{L \, }{2}} \right) \\ & - \frac{1 \, }{g} \, Be^{{ - \frac{\tau t}{2}}} \left( {\frac{\tau }{2}cos \, st - s \, sin \, st} \right)\left( {y - \, \frac{L \, }{2}} \right) \\ \end{aligned}$$

18 $$u\left( t \right) = Be^{{ - \frac{\tau t}{2}}} sin \, st$$

19 $$v\left( t \right) = - Be^{{ - \frac{\tau t}{2}}} {\text{cos }}st$$

where $s = \frac{1}{2}\sqrt {p^{2} - \tau^{2} }$ and $B$ is a constant.

In this simulation, the parameters were set as $L = 10000$ m, $h_{0} = 10$ m, $a = 3000$ m, and $B = 5$ m/s. The bed friction parameter was given by $\tau = 0.002\,s^{ - 1}$, relates to the Manning’s coefficient $n^{2} = \frac{{\tau h^{4/3} }}{{g\sqrt {u^{2} + v^{2} } }}$. The bed topography heights were defined with Eq. (16), while the initial water depth and velocities were defined by Eqs. 17–19, which is the analytic solutions at $t = 0$. The duration of the simulation is 6000 s, and performed with opened boundaries. In order to shows the performance of the simulation on the different grid size, the simulation is done on the domains have different number of grid cells, which are 50 × 50 cells, 100 × 100 cells, 200 × 200 cells, 400 × 400 cells and 800 × 800 cells.

**Fig. 4:**
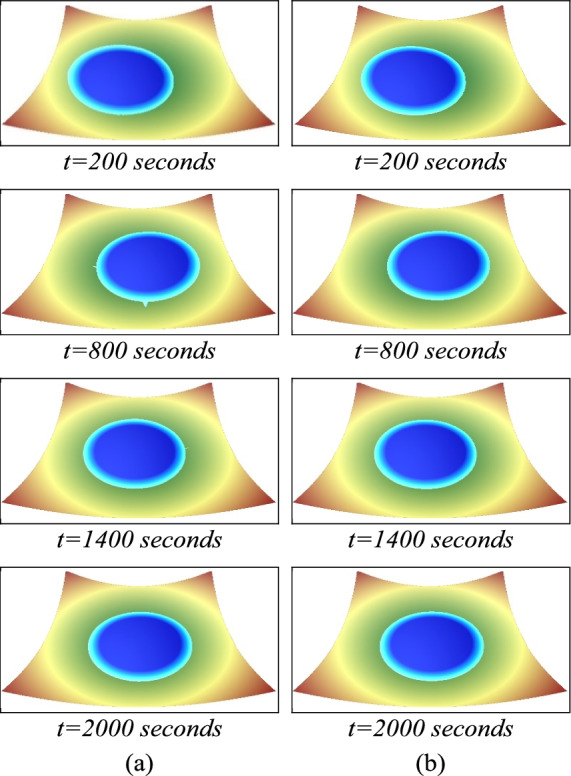
The numerical solutions (**a**) compared with the analytic solution (**b**) for the 2D parabolic bed topography simulation at different times.

The numerical results presented in Fig. 4 are simulations on the domain of 800 × 800 cells. The 3D visualization of water flow over parabolic bowl obtained by the numerical scheme are nearly the same as that obtained from the analytical solutions. Table 1, the results showed decrease in the value of RMSE as the number of grid cells increased. However, the simulation obtained by the proposed parallel algorithm takes less computational time than a simulation without the parallel algorithm. Especially when the number of grid cells increases and requires more time and computation, the parallel algorithm can greatly reduce the computational time of the simulation. However, for the results using the small number of computational cells (50 × 50 and 100 × 100), the parallel program takes more computational time than the serial program. The speed ratio demonstrates the growing efficiency of parallel processing for larger grids, shifting from negative values (e.g., −388.46% for 50 × 50) to substantial positive gains (e.g., 75.61% for 800 × 800).

**Table 1:** The results of the 2D parabolic bowl simulation on the different grid sizes obtained by the parallel program compared with those of the serial program.

No. of cells	Cell size (m^2^)	RMSE			No. of time steps	Computational times (s)		Speed ratio (%)
		h (m)	u (m/s)	v (m/s)		No parallel	parallel	
50 × 50	200 × 200	0.7001	6.5751	7.6555	656	2.479	12.109	− 388.46
100 × 100	100 × 100	0.365	6.5516	7.5934	1,296	24.041	36.938	− 53.65
200 × 200	50 × 50	0.1851	6.5374	7.5492	2,576	171.588	115.257	32.83
400 × 400	25 × 25	0.0929	6.5164	7.5134	5,138	1,339.423	501.064	62.59
800 × 800	12.5 × 12.5	0.0463	6.4903	7.4868	10,252	9,839.938	2,400.416	75.61

It nodes that rectangular grid cells may not perfectly match curved surfaces and can cause minor errors, especially on steep slopes. However, the Finite Volume Method (FVM) helps reduce these errors by calculating fluxes at cell boundaries. Higher grid resolutions (e.g., 800 × 800 cells) improve the representation of the bowl geometry, reducing RMSE values as shown in Table 1. Moreover, the use of a parallel algorithm significantly accelerates computations, enabling efficient processing of high-resolution grids and balancing accuracy with computational efficiency.

#### Dry dam break experiment simulation

**Fig. 5:**
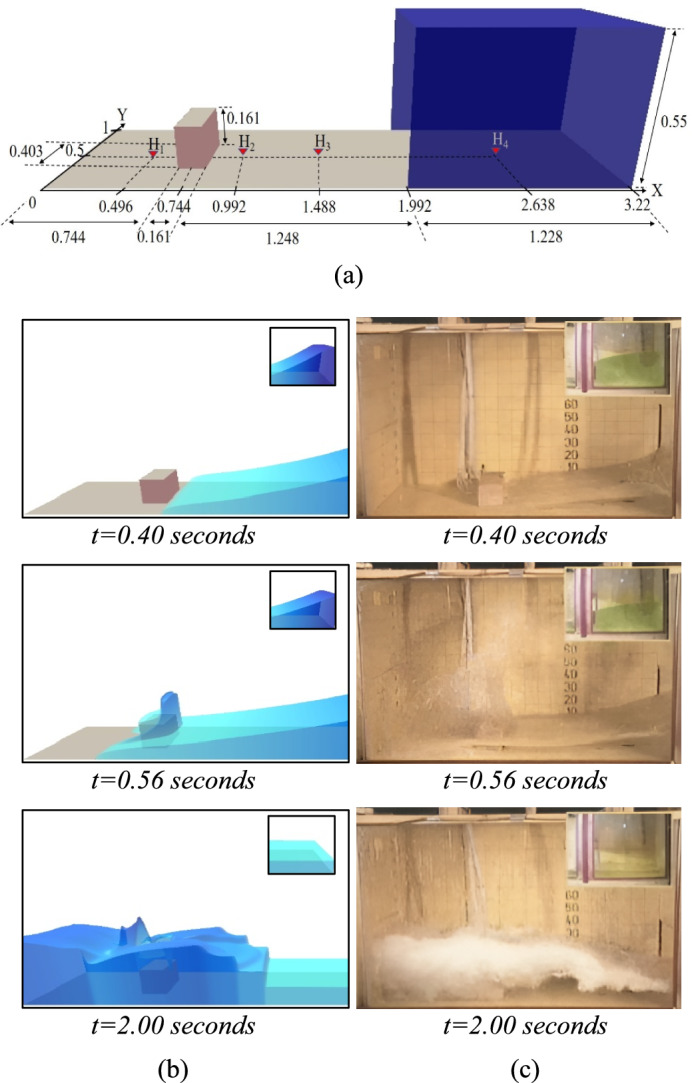
The geometry of the physical experiment of Kleefsman et al. (2005) and the locations of the verical wave probes $H_{1}$, $H_{2}$, $H_{3}$ and $H_{4}$ (**a**), the 3D representations of the dam break simulation (**b**) were compared with the experiment (**c**) at different times.

The numerical scheme simulation of a dam break with an obstacle was performed and compared with the experimental data of Kleefsman et al.^26^. The geometry of the physical experiment, where $H_{1}$, $H_{2}$, $H_{3}$ and $H_{4}$ are corresponding to the vertical wave probes which are set in the middle of tank as shown in Fig. 5a. The evolution of water depth was measured at each location and stored as data for model validation. This experiment was designed as a rectangular frame which closed tank. The gate was located at position *x* = 1.992 m and could be quickly removed to simulate the dam-break flow. The initial depth in front of the gate is 0.55 m, while the downstream is dry. The obstacle placed in the location *x* = 0.744 m and in the middle of the tank.

The experiment was simulating the dam break in the closed tank, the boundary condition will thus be considered as closed boundaries, i.e., there was no flow-out at the sides. The duration of the simulation was 6 s, during which the water flow crashed the boundary after the gate opened and returned to its initial location. This simulation was performed with Manning’s coefficient of 0.032.

The results illustrated in Fig. 5b and c were a simulation of the behaviour of water flow on the domain of 1200 × 400 cells. It showed the 3D visualization of the numerical results (b) compared with the real experiment (c). The numerical results showed that at 0.4 s, the dam-break flow was moving to the front of the obstacles. The water flow of the dam break moved towards the center of the obstacles, the dam break flow surrounding the obstacles, and formed vortexes at 0.56 s. Finally, the water flow reached the tank boundary, where it surged to crash into the obstacles at t = 2 s. The results displayed were very similar to those obtained in the experiment.

Table 2, the results showed a nearly constant value of RMSE as the number of grid cells increased. The simulation obtained by the proposed parallel algorithm used less computational time than a simulation without the parallel algorithm. As the number of grid cells increases, it used more time steps and computation time. However, the parallel algorithm can reduce the computational time of the simulation. The parallel program still used more computational time than the serial program for the small number of computational cells (75 × 25 and 150 × 50). The speed ratio demonstrates the growing efficiency of parallel processing for larger grids, shifting from negative values (e.g., −157.01% for 75 × 25) to substantial positive gains (e.g., 75.78% for 1200 × 400).

**Table 2:** The results of the dry dam break simulation on the different grid sizes obtained by the parallel program compared with those of the serial program.

No. of cells	Cell sizes (m^2^)	RMSE				No. of time steps	Computational times (s)		Speed ratio (%)
		H_1_(m)	H_2_(m)	H_3_(m)	H_4_(m)		No parallel	parallel	
75 × 25	0.0429 × 0.04	0.0375	0.0391	0.0244	0.0254	6,000	44.343	113.966	− 157.01
150 × 50	0.0215 × 0.02	0.0402	0.0428	0.0249	0.0250	6,000	153.593	155.963	− 1.54
300 × 100	0.0107 × 0.01	0.0395	0.0463	0.0247	0.0264	6,000	657.113	259.131	60.57
600 × 200	0.0053 × 0.005	0.0397	0.0446	0.0253	0.0265	8,938	3,496.614	1,055.973	69.80
1200 × 400	0.0027 × 0.0025	0.0436	0.0473	0.0263	0.0271	15,351	24,361.704	5,901.462	75.78

#### Wet dam break experiment simulation

**Fig. 6:**
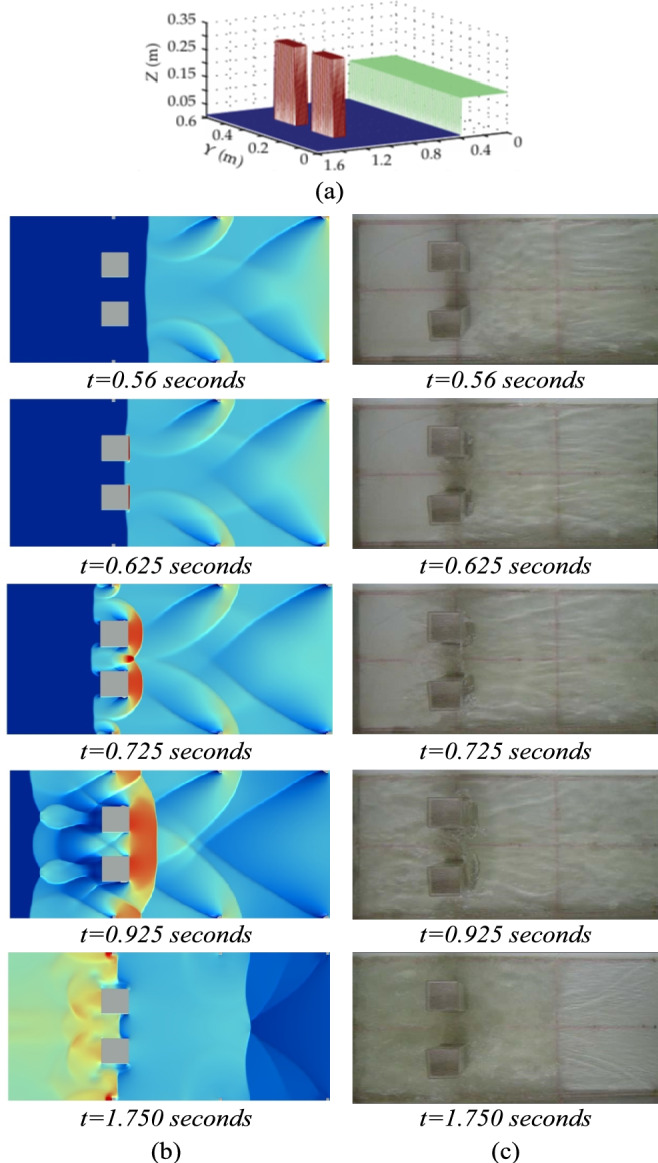
(**a**) The geometry of the wet dam break experiment from Peng, (2012), the numerical solutions (**b**) compared with the experiment (**c**) for the wet dam break simulation at different times.

An additional experiment with a wet dam break simulation was used to validate the parallel algorithm. The numerical scheme simulation of a dam break with an obstacle was performed and compared with the experimental data of Peng^27^. This experiment was designed as a rectangular frame with closed tank. The initial depth in front of the gate is 0.15 m, while the downstream area is wet with a water depth of 0.01 m as shown in Fig. 6a. Two square obstacles, the length and width being 0.1 m and the height being 0.3 m, are placed in the location *x* = 1.2 m and are paralleled placed in the middle of the tank with a distance of 0.1 m, Moreover, six small obstacles are paralleled and placed on both side walls at positions *x* = 0.4 m, 0.8 m, and 1.2 m in order to mimic the natural channel not being as flat as the artificial construction.

The experimental results illustrated in Fig. 6b and c were the simulation of the behavior of water flow at five difference time on the domain 1200 × 400 cells. It showed the 2D visualization of the numerical results (b) compared with the experiment (c). The numerical results are visualized using a color gradient representing water depth. The results demonstrated that the numerical model accurately captured the behavior of the water flow as it crashed into obstacles and formed surges, including the movement of the flow due to small obstacles, which was consistent with the experiment.

Table 3 presented the performances of the parallel algorithm. The results showed that the parallel algorithm takes less computational time than a simulation without the parallel algorithm. As the number of grid cells increase, using more time steps and computation time, the parallel algorithm can greatly reduce the computational time of the simulation. Similar to the results before, for the small number of computational cells (80 × 30), the parallel program used more computational time than the serial program. The speed ratio demonstrates the growing efficiency of parallel processing for larger grids, shifting from negative values (e.g., −134.00% for 80 × 30) to substantial positive gains (e.g., 75.07% for 1280 × 480).

**Table 3:** The results of the wet dam break simulation on the different grid sizes obtained by the parallel program compared with those of the serial program.

No. of cells	Cell sizes (m^2^)	No. of time steps	Computational times (s)		Speed ratio (%)
			No parallel	Parallel	
80 × 30	0.02 × 0.02	400	3.532	8.265	− 134.00
160 × 60	0.01 × 0.01	800	28.180	24.191	14.16
320 × 120	0.005 × 0.005	1,508	206.758	73.481	64.46
640 × 240	0.0025 × 0.0025	2,907	1,570.515	394.825	74.86
1280 × 480	0.00125 × 0.00125	5,824	12,659.016	3,156.47	75.07

#### The Xe-Pian Xe-Namnoy dam break simulation

**Fig. 7:**
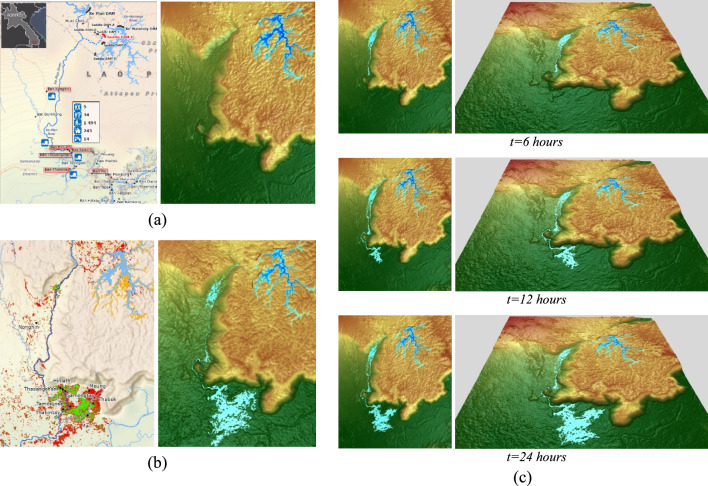
(**a**) The location of the Xe-Pian Xe-Namnoy dam in Laos (left) and the intial dam break simulation (right), (**b**) The comparison of the satellite detected flood water (left) and the simulation result (right), and (**c**) The 2D (left) and 3D (right) visualization of the dam-break flood simulation at different times.

In this section, we used numerical algorithms to simulate the dam break flow on natural topography, specifically in the water flow after the Xe-Pian Xe-Namnoy dam break that occurred in Laos on 23 July 2018. The study area covers 320.76 km^2^ from 14.54018481936183^o^N to 15.16877265406895^o^N, and from 106.2761485796276^o^E to 106.7516188185062^o^E. The simulation is performed using the digital terrain data (51,480 m × 68,040 m), obtained from the NASA Shuttle Radar Topography Mission (SRTM) data source in a Digital Elevation Model (DEM) with a resolution of 90 × 90 m (http://srtm.csi.cgiar.org). The dam break flood was simulated on the initial grid size of 1144 × 1512 cells with resolution of 45 × 45 m, obtained via the bilinear interpolation. The initial water level of the reservoir behind the dam was defined 805 m. The initial water discharges in the *x* and *y* directions were set to be zero, while the boundary condition was defined as the opened boundaries. The location of the Xe-Pian Xe-Namnoy dam (The image from THAIWATER.net, https://tiwrm.hii.or.th) and the initial dam break simulation are presented in Fig. 7a. The duration of the simulation is the first 52-h period of the dam break flood event.

Manning’s coefficient was assumed to be 0.035 as a uniform value representing the average roughness across all points in the simulated area. This value was determined through a trial-and-error approach, where Manning’s roughness coefficient was tested within a typical range of 0.01 to 0.05, suitable for floodplains with vegetation and uneven terrain. Multiple simulations were conducted using different Manning’s values, and the results were systematically compared against the actual flood extents observed in satellite imagery of the real flood event. The value of 0.035 was identified as optimal, as it provided the closest match between the simulated flood areas and the inundation patterns captured by satellite images. The simulation involved the sudden removal of the dam structure, a common approach in dam break modeling to represent worst-case scenarios and assess the robustness of numerical schemes. While real dam break scenarios include additional complexities like spillways, weirs, and gradual erosion processes, this study focused on hydrodynamic behavior following an instantaneous dam failure.

Figure 7b shows the simulation results compared with the satellite image at 52-h after the dam break event. The simulation of flooding area is nearly the same as that obtained from the satellite detected flood water from THAIWATER.net (https://tiwrm.hii.or.th). The 2D and 3D visualization of the simulation are presented at different points in time in Fig. 7c. To evaluate the accuracy of the simulation, a classification map was created by overlaying the simulation results with the observed satellite imagery. This classification map served as the basis for constructing a confusion matrix, which quantifies the agreement between the modeled and observed flood extents. The confusion matrix consisted of four key components: True Positives (TP), representing areas correctly predicted as flooded and confirmed by satellite imagery; False Positives (FP), indicating areas incorrectly predicted as flooded but not observed as such; False Negatives (FN), referring to areas missed by the model but observed as flooded; and True Negatives (TN), denoting areas correctly predicted as non-flooded and confirmed by satellite imagery. The values obtained were TP = 16,785, FP = 5705, FN = 11,077, and TN = 31,960. From these values, several metrics were calculated based on standard evaluation formulas in^28^. Accuracy, which measures the overall correctness of predictions, was computed as Accuracy = (TP + FP)/(FN + TN + TP + TN) ≈ 0.7439, indicating that approximately 74.39% of all predictions were correct. Precision, representing the proportion of correctly predicted flooded areas among all predicted floods, was Precision = TP/(FP + TP) ≈ 0.7463, showing that when the model predicts a flood, it is correct about 74.63% of the time. Recall, which indicates the ability to correctly identify actual flooded areas, was Recall = TP/(FN + TP) ≈ 0.6024, meaning that the model correctly identified 60.24% of the actual flooded areas. Specificity, measuring the proportion of correctly predicted non-flooded areas among all actual non-flooded areas, was Specificity = TN/(FP + TN) ≈ 0.8485, indicating that the model correctly identified approximately 84.85% of the actual non-flooded areas. Finally, the F1-Score, a balanced measure of precision and recall, was calculated as F1-Score = 2 × Precision × Recall/(Precision + Recall) ≈ 0.6667, providing a balanced measure between precision and recall. The results derived from the confusion matrix values demonstrate reasonable accuracy. However, differences were observed between the modeled and observed flood areas. These differences likely occurred because the model did not account for buildings. In some places, the actual flooded areas were larger than predicted since buildings pushed water outward, spreading it further than the model expected. In other areas, the model wrongly showed flooding where there was none, as these areas had elevated buildings that stayed above the water level. The efficiency of the parallel technique was evaluated by comparing the results of simulations computed by the parallel program to those computed by the serial program (without the parallel technique). Table 4, the results show that the parallel program can help reduce computational times by performing of speed ratio up to 65.66% faster than the serial program.

**Table 4:** The results of the dam break simulation obtained by the parallel program compared with those of the serial program.

No. of grid cells	Cell size (m^2^)	No. of computed cells	No. of dry cells	No. of time steps	Computational times (s)		Speed ratio (%)
					No parallel	Parallel	
1144 × 1512	45 × 45	74,061	11,783	94,343	17,063.796	5,859.421	65.66

## Discussions and conclusions

This paper proposed the parallel program by applying the ADU method for solving the shallow water equation with the well-balanced positivity-preserving first order finite volume scheme. The numerical simulations show that the developed algorithm is suitable for simulating and visualizing the water flow, which agrees closely with other results obtained in the literature and for both experimental and theoretical results. This shows the accuracy of the proposed parallel program. In terms of performance, the proposed parallel program’s computational time was tested and compared with that of the serial program, which used only the ADU method without the parallel technique. The parallel program helps reduce computational time while maintaining the same accuracy as the serial program. The performance improvement can be attributed to the efficient use of multi-core processors, which allowed simultaneous computation of independent cells, a capability that is absent in the serial program. In contrast, the serial program processes each cell sequentially, leading to significantly higher computational time, especially as the domain size increases. Additionally, the parallel index-array method reduces the complexity of programming and algorithm design by minimizing communication overhead between cores. Instead of frequently exchanging boundary information, as required in traditional grid-based parallelism, this method directly accesses neighboring cell data. This streamlined approach simplifies the implementation of inter-core communication, making it particularly advantageous when combined with the ADU method, which dynamically adjusts the computational domain over time. By avoiding complex data exchanges, the method not only enhances computational efficiency but also reduces the challenges associated with designing parallel algorithms.

However, it can be noted that using the parallel program for the simulation with a small number of computational cells does not necessarily mean good performance, and it may take more computational time than using the serial program. Moreover, although the use of a first-order finite volume scheme is simple and stable but limited for simulating floods in steep areas. It cannot accurately capture fast changes in water flow or sudden depth variations. This needs very fine grids to improve accuracy, making it more time-consuming and expensive. In the serial program by Busaman et al.^29^, adaptive mesh refinement (AMR) combined with a second-order finite volume scheme was applied to solve SWEs. The results demonstrate that this approach significantly enhances flow profile accuracy, achieving outcomes similar to those obtained using the finest grid, whereas coarse grid simulations produce unacceptable results. Future research should develop parallel algorithms for high-order schemes, and use AMR to enhance accuracy and efficiency in flood simulations.

Although a direct comparison with the results of Tanaka et al.^13^ could not be conducted, the findings in this study demonstrate that integrating the ADU method with parallel computing effectively reduces computational time, consistent with their reported outcomes. However, the use of the full SWEs may offer advantages over the simplified SWEs. This is because the simplified SWEs neglect momentum terms, such as the advection term, while the full SWEs retain these terms. The retained advection term is essential for accurately modeling complex flow behaviors, such as rapid water level changes and interactions with uneven terrains, especially in fast-moving water^30,31^. Comparative tests of Almeida et al.^30^ demonstrated that the simplified SWEs, when evaluated against the analytical solution of the full SWEs, exhibited slower wave propagation speeds and reduced accuracy in dynamic flow conditions, underscoring the limitations of neglecting the advection term. Additionally, Saiduzzaman and Ray^31^ found that numerical schemes preserving the advection term provided higher accuracy in simulating dynamic water flows, further supporting the use of full SWEs. This distinction makes the full SWEs more suitable for real-world flood modeling scenarios where dynamic flow interactions are significant. Future studies will comprehensively compare full and simplified SWEs formulations, testing their performance under various conditions using a parallel computing framework for efficient large-scale urban flood.

The parallel algorithm with the ADU method can be applied to solve water flow equations in various other flooding simulations on natural topography, such as dam breaks, river floods, flood inundations in cities, and utilization for the water management system, including parameter estimation in water flooding models. For dam break, the algorithm can be applied to simulate sudden water flows caused by dam breaks by initializing specific parameters and input conditions tailored to the target area. For river floods, the method can integrate with a one-dimensional river model that uses cross-sectional data to simulate flow within the river channel. When the flood extends beyond the riverbanks and covers a larger area, the parallel algorithm combined with the ADU method helps accelerate the computations significantly without compromising accuracy. For urban flooding, The simulation can be performed utilize polygon data of buildings, such as those from Google Maps, as input for simulations. The numerical method is well-suited for handling flow around obstacles, which can represent buildings and infrastructure in urban areas. By leveraging the ADU algorithm, unnecessary computations within non-flooded building zones can be omitted, optimizing the process. As the flooded area grows, the parallel algorithm dynamically handles the increased computational load efficiently. For applications in water management, the parallel algorithm and ADU method are invaluable for water management systems by reducing the computational time needed to simulate various scenarios. This allows for the collection of detailed flow information that can be used to develop optimization models for effective water management strategies. Additionally, for parameter estimation in flood models requiring the testing of multiple parameters sets, such as through genetic algorithms, the parallel algorithm and ADU method enable faster simulations. This accelerates the process of collecting flow information and identifying optimal parameter values for the model. Moreover, field-surveyed data, such as that seen in^32^, can support model validation and improve flood simulation accuracy by integrating real-time sensor readings and high-resolution elevation data into the modeling process​.

 These features make it a reliable and practical tool for solving real-world water flow and flooding problems. A comparison of the present work with other experts’ findings can be conducted in future research to ensure comprehensive validation and benchmark the approach against established methodologies.

## Data Availability

The datasets generated and/or analysed during the current study available from the corresponding author on reasonable request.
